# SMF-1, SMF-2 and SMF-3 DMT1 Orthologues Regulate and Are Regulated Differentially by Manganese Levels in *C. elegans*

**DOI:** 10.1371/journal.pone.0007792

**Published:** 2009-11-18

**Authors:** Catherine Au, Alexandre Benedetto, Joel Anderson, Arnaud Labrousse, Keith Erikson, Jonathan J. Ewbank, Michael Aschner

**Affiliations:** 1 Department of Pediatrics, Vanderbilt University, Nashville, Tennessee, United States of America; 2 Center for Molecular Toxicology, Vanderbilt University, Nashville, Tennessee, United States of America; 3 Children's Hospital, Vanderbilt University, Nashville, Tennessee, United States of America; 4 Department of Nutrition, The University of North Carolina Greensboro, Greensboro, North Carolina, United States of America; 5 Centre d'Immunologie de Marseille-Luminy, Université de la Méditerranée, Marseille, France; 6 U631, INSERM, Marseille, France; 7 UMR6102, CNRS, Marseille, France; 8 London Centre for Nanotechnology, University College London, London, United Kingdom; 9 Institut de Pharmacologie et de Biologie Structurale, Université Paul Sabatier Toulouse III, Toulouse, France; University Medical Center Groningen, Netherlands

## Abstract

Manganese (Mn) is an essential metal that can exert toxic effects at high concentrations, eventually leading to Parkinsonism. A major transporter of Mn in mammals is the divalent-metal transporter (DMT1). We characterize here DMT1-like proteins in the nematode *C. elegans*, which regulate and are regulated by Mn and iron (Fe) content. We identified three new DMT1-like genes in *C. elegans*: *smf-1*, *smf-2* and *smf-3*. All three can functionally substitute for loss of their yeast orthologues in *S. cerevisiae*. In the worm, deletion of *smf-1* or *smf-3* led to an increased Mn tolerance, while loss of *smf-2* led to increased Mn sensitivity. *smf* mRNA levels measured by QRT-PCR were up-regulated upon low Mn and down-regulated upon high Mn exposures. Translational GFP-fusions revealed that SMF-1 and SMF-3 strongly localize to partially overlapping apical regions of the gut epithelium, suggesting a differential role for SMF-1 and SMF-3 in Mn nutritional intake. Conversely, SMF-2 was detected in the marginal pharyngeal epithelium, possibly involved in metal-sensing. Analysis of metal content upon Mn exposure in *smf* mutants revealed that SMF-3 is required for normal Mn uptake, while *smf-1* was dispensable. Higher *smf-2* mRNA levels correlated with higher Fe content, supporting a role for SMF-2 in Fe uptake. In *smf-1* and *smf-3* but not in *smf-2* mutants, increased Mn exposure led to decreased Fe levels, suggesting that both metals compete for transport by SMF-2. Finally, SMF-3 was post-translationally and reversibly down-regulated following Mn-exposure. In sum, we unraveled a complex interplay of transcriptional and post-translational regulations of 3 DMT1-like transporters in two adjacent tissues, which regulate metal-content in *C. elegans*.

## Introduction

Manganese (Mn) is one of the most abundant natural elements in the Earth's crust. It most frequently occurs in the form of oxides, carbonates and silicates [1]. It is one of 7 essential metals for animals, acting as a cofactor for multiple proteins with a wide variety of biological activities, such as transferases, hydrolases, lyases, arginase, glutamine synthetase, superoxide dismutase and integrins [2], [3]. Consequently, Mn is essential for many physiological processes, such as modulation of the immune system, stellate process production in astrocytes, as well as protein, lipid and carbohydrate metabolism [4], [5], [6], [7], [8]. Mn is also essential for optimal brain and skeletal structure development [9], [10]; Mn deficiency has been associated with defective bone formation and increased susceptibility to seizures [11], [12]. Despite being essential for metabolic functions, Mn at high concentrations can be toxic, especially to the brain. Though most Mn is obtained through the diet, Mn toxicity from dietary intake is rare, because Mn balance is tightly regulated by both the enterocytes (intake) and the biliary duct cells (excretion). In contrast, pulmonary uptake and particulate transport via the olfactory bulb [2], [13], [14] can lead to deposition of Mn within the striatum and cerebellum, and inflammation of the nasal epithelium [15]. Exposure to excessive Mn levels may cause an extrapyramidal syndrome, referred to as manganism, closely resembling idiopathic Parkinson's disease (IPD), both at the molecular and clinical level [16], [17], [18]. Mn exposure represents a significant public health matter due to the use of Mn as a catalyser in countless industrial processes, its presence in gasoline additive, fungicides such as Maneb and in permanganate, a drinking water purifier [1], [2], [19], [20], [21]. Because Mn is the only environmental toxicant that has been robustly associated with IPD, studies on the mechanisms that mediate its toxicity offer means for better understanding of neurodegenerative diseases, the frequency of which is on the rise [22], [23], [24].

Due to the delicate relationship between essentiality and toxicity, Mn homeostasis is crucial for all eukaryotes. Previous research has focused on Mn transport mechanisms across the blood-brain barrier (BBB), but the nature and relative contributions of the carrier(s) identified thus far remain controversial. Over the past two decades, active transport [25] and facilitated diffusion [26], [27] mechanisms have been described. More recently, Mn transport has been ascribed to high affinity metal transporters of calcium (Ca) and iron (Fe). Amongst these, attention has been directed to the divalent metal transporter (DMT1 [28]), which belongs to the family of natural resistance-associated macrophage protein (NRAMP) [29], [30], [31], [32]. To avoid confusion, we will further refer to NRAMP family members as DMT(s) for Divalent Metal Transporter(s).

DMT1, previously known as NRAMP2, was first identified as an orthologue of NRAMP1, a protein involved in host defense against several types of infection [29], [30]. Subsequently, it was referred to as divalent cation transporter (DCT1), because of its ability to transport divalent zinc (Zn^2+^), manganese (Mn^2+^), cobalt (Co^2+^), cadmium (Cd^2+^), copper (Cu^2+^), nickel (Ni^2+^), lead (Pb^2+^), and iron (Fe^2+^) [32], [33], [34]. In 1999 it was designated as DMT1 [28]. DMT1 is an integral membrane protein conserved from bacteria to humans, containing 11 to 12 transmembrane domains (TMD) and a “consensus transport sequence” (CTS) involved in divalent metal ion transport [32], [33], [34], [35]. Notably, rodent models presenting a spontaneous mutation in *DMT1*, microcytic (mk) mice and Belgrade (b/b) rats [36], and several human mutations in *DMT1* [37], [38], [39], [40], [41], [42] suggest an association between microcytic anemia, impairment of Fe transport and Mn homeostasis. In vertebrates, DMT1 is ubiquitously expressed, but more abundant expression is observed in the proximal duodenum compared to the kidney or the brain [28], [32], [43]. Within the brain, the basal ganglia express higher levels of DMT1, where Mn preferentially accumulates [44]. At a subcellular level, DMT1 is strongly localized at the apical membrane in enterocytes [45] and sustentacular cells of the olfactory epithelium [14] whereas in macrophages, it is restricted to the phagosomal membrane. The vertebrate *DMT1* gene produces four alternatively spliced mRNAs differing by the presence or absence of the exon 0, and a 3′ sequence in which an iron regulatory element is present or absent (+IRE and -IRE respectively). The 3′ UTR is responsible for the modulation of the +IRE mRNA stability by the intracellular Fe pool [32]. The +IRE vs -IRE mRNAs encode DMT1 isoforms with distinct carboxy- termini [43], [46]. The +IRE isoforms are found mainly at the apical membrane of epithelial cells [45], [47] and in late endosomes and lysosomes within HEp-2 cells [40], [48]. The -IRE isoforms are found predominantly in early and recycling endosomes [49], [50], [51]. Studies in yeast have identified three DMT1 orthologues: Smf1p, Smf2p and Smf3p, encoded by *SMF1, SMF2* and *SMF3* respectively. In contrast to vertebrate *DMT1*, no typical IRE is found in any of the *SMF* genes, and the poor sequence conservation of the C-termini is not predictive of a correspondence between the vertebrate –IRE/+IRE isoforms and Smf1p/2p/3p. However, Smf1p was identified as a non-specific metal ion transporter for Mn^2+^, Zn^2+^, Cu^2+^, Fe^2+^ and Cd^2+^ [52], [53] and Smf2p was also found to be involved in Mn transport [54]. Analogous to their vertebrate orthologues, the yeast DMTs are found in various intracellular compartments: Smf1p at the cell surface, Smf2p in vesicles and Smf3p exclusively at the vacuolar membrane [53], [55], [56]. In addition, they differentially contribute to Mn homeostasis, since Smfp1 and Smf3p are dispensable in Mn-replete conditions [53], while Smf2p is essential for ensuring proper Mn uptake [57], [58]. Additionally, the *SMF3* promoter is found to contain a target sequence for the Fe-sensing transcription factor Aft1p, which is responsible for its transcriptional regulation by Fe, whereas Smf1p and Smf2p levels are unaffected by Fe concentration [55], [56].

Currently, both Mn transport and DMT protein function(s) are poorly understood. This is partly due to the existence of several DMT proteins differentially regulated at the transcriptional and post-translational levels, in distinct tissues (intestine, liver, kidney, brain) and under various conditions (Mn or Fe levels, infection). Moreover, findings in a unicellular organism like the baker's yeast are difficult to translate to metazoans and especially to mammals. Given the current lack of a genetically amenable animal model, we used the *C. elegans* system to address these issues. We identified and cloned three functional *C. elegans* DMT1 orthologues SMF-1, SMF-2 and SMF-3, with distinct roles in Mn transport regulation. Our results support an evolutionary conserved function for DMT1 isoforms in the regulation of Mn uptake, and emphasize the differential contribution and regulation of DMT1 isoforms expressed in different tissues and exhibiting different subcellular localizations. Furthermore, our study identifies SMF-3 as the main Mn uptake transporter in the worm, whereas SMF-1 has a minor role in this process and SMF-2 is involved in metal content regulation.

## Methods

*Multiple alignment generation and analysis. -* Protein sequences were uploaded from the NCBI website (http://www.ncbi.nlm.nih.gov/sites/entrez). Multiple alignments and phylogenetic trees were generated using ClustalX1.81 [59] running ClustalW [60].

*Yeast handling, transformation and EGTA sensitivity assessment. -* Yeast strains, maintenance, transformation and culture for the EGTA sensitivity assay were performed as described [58]. Plasmids pVT-Cesmf-1, pVT-Cesmf-2 and pVT-Cesmf-3, for yeast expression of *C. elegans* genes *smf-1*, *smf-2* and *smf-3* respectively, were generated as follows. *smf-1* (from yk452d4) and *smf-3* (from yk397h3) cDNA were kindly provided by Yuji Kohara, while *smf-2* cDNA was obtained by reverse-transcription-PCR. *smf-1* was amplified using primers 5′-CAG CGG ATC CGC TTG ATA TCC TGC ATT GTC-3′ and 5′-GAC CGG TAC CGG AAA GTA TAC ATC GTT CAC-3′. *smf-3* was amplified using primers 5′-CGCGGATCCAATGGAGGTGAAATGAAAT-3′ and 5′-GCCCGGTACCGCATATCGCATAGACAGTTC-3′. *smf-1*' *smf-2*, and *smf-3* fragments were then cloned into pVT101-U using HindIII and PstI.

C. elegans *strains and handling of the worms. - C. elegans* strains were handled and maintained at 20°C as previously described [61]. The following strains were used: N2 (wildtype); IG6, *smf-1(eh5) X*; VC171, *smf-2(gk133) X*; RB1074, *smf-3(ok1035) IV*; MT455, *lon-2(e678) unc-18(e81) X*; MT628, *dpy-9(e12) unc-17(e245) IV*. All strains were provided by the Caenorhabditis Genetic Center (CGC, Minnesota). The IG6, VC171 and RB1074 strains were outcrossed 4 additional times to generate MAB23, MAB21 and MAB37, respectively.

*Gene cloning and plasmid constructions. -* Restriction enzymes were ordered from New England Biolabs. PCR amplifications used the LA Taq™ Polymerase (Takara), and were performed on an Eppendorf2 PCR-machine. Translational C-terminal GFP-fusions for SMF-1, SMF-2 and SMF-3 localization were generated as follow. The *smf-1* ORF and preceding intergenic region (4.4 kb) was amplified using primers: 5′- TAT TAC CTG CAG GAG CTA GCT TCA TGT TCA CCG CCA AGC TCG-3′, and 5′-TAT TAG GAT CCA ATT GAT ATC CTG CAT TGT CAT GGA CTGC-3′. This fragment was cloned into the pPD95.75 vector (Fire kit) between the SbfI and BamHI restriction sites, in frame with the GFP (plasmid pMA0015). The *smf-2* ORF with its preceding intergenic sequence (3.5 kb) was amplified using primers: 5′-TAT TCT GCA GTC ATA CGA AAA CGA TGC TCC GTG-3′ and 5′-TAT TGG TAC CAC AAA GTA TAC ATC GTT CAC AAC-3′. The *smf-3* ORF and its preceding intergenic region (8.4 kb) were amplified using primers: 5′-TAT TCT GCA GAC TTC ATT GGG GAT GTG CTT TGG-3′ and 5′-GGT ACC CAA TAT CGC ATA GAC AGT TCG TCG-3′. The *smf-2* and *smf-3* amplification products were then cloned into pPD95.75 between the PstI and KpnI restriction sites, in frame with the GFP (plasmid pMA0010 and pMA0004 respectively). Transcriptional constructs for *smf-1*, *smf-2* and *smf-3* were obtained by cloning the preceding intergenic region, respectively up to the 2^nd^, 6^th^ and 7^th^ exon to include highly conserved intronic sequences. The following primer sets were used: 5′-CGC AAG CTT CGA GCA GCT CCG ATT G-3′ and  5′-CGC CTG CAG CCT TGT GCG CCA GAC TGA AGG-3′ for *smf-1*, 5′-TAT TGC ATG CTC ATA CGA AAA CGA TGC TCC GTG-3′ and 5′-TAT TCT GCA GTA GTC CAA ACT GAC ATC CCA GG-3′ for *smf-2*, 5′-TAT TCT GCA GAC TTC ATT GGG GAT GTG CTT TGG-3′ and 5′-TAT TGT CGA CGG CTC TGG AAT ATA ATT AGG ATT GC-3′ for *smf-3*. The *smf-1* product (1.7 kb) was cloned between the HindIII and PstI restriction sites in pPD95.75 (promSmf1GFP1). The *smf-2* (2.6 kb) fragment was cloned into pPD95.69 (Fire kit) between the SphI and PstI restriction sites (pMA0009). The *smf-3* (5.5 kb) fragment was cloned into pPD95.69 between the restriction sites PstI and SalI (pMA0003).

C. elegans *transgenesis. -* DNA was injected into the syncytial gonads of N2 hermaphrodites using injection mixes containing 25 ng/µl construct and 175 ng/µl pRF4 as a transformation marker [62]. A minimum of three independent transgenic strains per construct showing the same expression pattern were observed. One strain for each construct was selected for this study: MAB111, *mjaEx074[SMF-1::GFP; rol-6(su1006)]*; MAB120, *mjaEx083[SMF-2::GFP; rol-6(su1006)]*; MAB105, *mjaEx068[SMF-3::GFP; rol-6(su1006)]*.

*Acute manganese chloride treatments. -* Manganese chloride (MnCl_2_) solutions were prepared in 85 mM NaCl. For each tested strain, 5000 synchronized L1 worms per tube were exposed to 0 to 4 M of MnCl_2_ in siliconized tubes for 30 minutes. Each condition was performed in tetraplicates. Worms were then pelleted by centrifugation at 7000 rpm for 3 minutes and washed 5 times in 85 mM NaCl. Worms from each of the siliconized tubes were then transferred into individual 100 mm OP50-1 coated NGM plates, which were then blinded. At 24 h post-treatment, for each plate, live worms were scored in 4 random 1 cm^2^ grids to estimate the total number of surviving worms (up to 800 counts per plate). Scores were normalized to percent control (0 mM MnCl_2_ exposure).

*Mn and Fe content measurement by atomic absorption spectrophotometry (AAS). -* Triplicates of 7000 L1 worms per condition were treated with MnCl_2_ as previously described. The samples were washed 8 times in 85 mM NaCl. The samples were dehydrated in a vacuum oven at 65°C for 2 hours, and further digested in 200 µl ultrapure nitric acid for 24 hours in a sand-bath (60°C). A 20 µl aliquot of the digested sample was brought to 1 ml total volume with 2% nitric acid and analyzed for Mn and Fe content using graphite furnace atomic absorption spectroscopy (AAS) (Varian AA240, Varian, Inc USA). Bovine liver digested in ultrapure nitric acid was used as an internal standard for analysis (NBS Standard Reference Material, USDC, Washington, DC, diluted at 5 µg Mn/L and 92 µg Fe/L).

*Epifluorescence, DIC and confocal microscopy. -* For each slide, at least 30 worms were mounted on 4% agarose pads in M9, and anaesthetized with 0.2% tricaine/0.02% tetramisole in M9. Fluorescence observations and DIC imaging were performed with an epifluorescence microscope (Nikon Eclipse 80i, Nikon) equipped with a Lambda LS Xenon lamp (Sutter Instrument Company) and Nikon Plan Fluor 20x dry and Nikon Plan Apo 60x 1.3 oil objectives. The microscope was coupled to a black-and-white camera (DS-Qi1Mc; Nikon) operated by the Nikon Elements AR3.0 software (NES AR3.0) for image acquisitions. Confocal images acquired for illustration or GFP intensity measurement purposes were captured through Plan-Neofluar 40×, Plan-Apochromat 63x, or Plan-Neofluar 100× oil objectives with a 1.3, 1.4 and 1.3 apertures, respectively, on a LSM510 confocal microscope (Carl Zeiss MicroImaging, Inc.), scanning every 200 nm for XZ sections. Images were processed with the Zeiss LSM Image Browser 4.0.0.157 software and edited using Photoshop 7.0 (Adobe). Microscopes were in air-conditioned rooms (20–22°C).

*Phenotypic characterization of Mn treated worms.-* Following Mn treatment up to 24 h post-treatment, worms were observed under stereomicroscope (Zeiss), and mounted for microscopy phenotypic analysis using our Nikon platform aforementioned. After 24 h at 20°C, at least 30 random control worms and 30 treated worms exposed to 35 mM MnCl_2_, were imaged at 20x. We assessed their developmental stage (L1 or L2) and their size using NES AR3.0.

*SMF-3::GFP fluorescence measurements. -* SMF-3::GFP transgenic worms (MAB105) were acutely treated as described previously, transferred on OP50-1 seeded NGM plates and imaged at 1 h, 5 h and 30 h post-treatment. Fluorescence measurements of SMF-3::GFP signal were performed on complete confocal Z-stack projections of *C. elegans* gut. 5 to 12 animals of the same age were imaged for each condition. Treated and untreated animals were mounted on the same slide, and imaged with the same magnification, gain, offset, pinhole and laser power settings. Due to the very small signal/noise ratio for treated animals at 5 h, those settings allowed up to 30% signal saturation for some of the 5 h, 30 h control worms and 30 h recovering worms, so that fluorescence measurements for these conditions are underestimates. Mean signal intensity of the maximal projection of the apical membrane of the intestine (from the pharyngeal-intestinal valve to the rectum) was measured using the freeware ImageJ.

*Statistics. -* Dose-response lethality curves and histograms for Mn content measurements were generated using GraphPad Prism (GraphPad Software Inc.). We used a sigmoidal dose-response model with a top constraint at 100% to draw the curves and determine the LD_50_ values. Statistical analysis of significance was carried out by one-way ANOVA for the dose-response curves, and two-way ANOVA for Mn content measurements followed by post-hoc Bonferroni test when the overall p value was less than 0.05. For Fe content, Fisher test revealing differences in variances between the groups, two-tailed unpaired T-test with Welch's correction were used to assess differences in Fe content. In all figures, error bars represent SEM, * p<0.05, ** p<0.01, *** p<0.001.

*RNA isolation, cDNA preparation and Real-time PCR. -* 10000 N2, MAB21, MAB23 and MAB37 synchronized L1 worms per tube were acutely treated with MnCl_2_ concentrations of 0, 0.1, 10 and 100 mM, washed in NaCl 85 mM, pelleted and frozen in liquid nitrogen. They were thawed on ice, frozen again in N_2liq_ and resuspended in 100 µL H_2_O and 350 µL Trizol (Invitrogen Life Technologies) for RNA isolation. RNA extracts were purified using Qiagene RNeasy mini-kit (Qiagen). Messenger RNAs were reverse-transcribed with oligo-dT primers using the Superscript III RNAse H Reverse transcription kit (Invitrogen). cDNAs were stored at −20°C. Quantitative Real-time-PCR was carried on a ABISystems HT7900, using Brilliant SYBR Green I kit (Stratagene/Agilent Technologies Inc.), and HPLC purified primers (Operon) at a final concentration of 120 nM. For amplification of *smf-1* cDNA, two sets of 22b primers were designed over the last intron 5′-GCT CCG ATC ACC TTT GCA TAC G-3′/5′-ATC CTC GGA TGG AAA CGG TGT C-3′, and between exons 6 and 8 5′-TTT CGC ACA TGG ACT TTA CCA G-3′/5′- GCA ATA GCT CCA AAC TGG CAT C-3′. For *smf-2*, primers over intron 6 5′-TAT TCG CAG CAG GAC AAT CAT C-3′/5′-TTG TGC ATA ATC CGC TTA CTG G-3′, and over intron 10 5′-GTT GCT TGC GAA CTT ATG AAC G-3′/5′-ACA AAG GTT TCT GTG ATC CAC G-3′, were used. Smf-3 cDNA was amplified using primer sets 5′-TCC AGT GCT GAC ATT TGT ATC G-3′/5′-CAA GGA AAT CAC AAT GGA GAC C-3′ over intron 10 and 5′-GGT CTC CAT TGT GATT TCC TTG-3′/5′-CGA AAT CGT GGT AGA TGG GCT CC-3′ over intron 11. *cdc-42* (primers: 5′-CTG CTG GAC AGG AAG ATT ACG-3′/5′-CTC GGA CAT TCT CGA ATG AAG-3′) and *Y45F10D4F.3* (primers: 5′-GTC GCT TCA AAT CAG TTC AGC-3′/5′-GTT CTT GTC AAG TGA TCC GAC A-3′), were used as controls to normalize *smf* expression levels as they were proven to have stable expression levels independent of age or stress conditions [63]. The amplification setup included 10′ denaturation at 95°C, 20 cycles at 95°C for 30″/58°C for 1′/70°C for 1′, 20 to 25 cycles at 95°C for 30″/56°C for 45″/72°C for 45″, and a dissociation curve to ascertain that the signal did not result from primer annealing.

## Results

**Figure 1:**
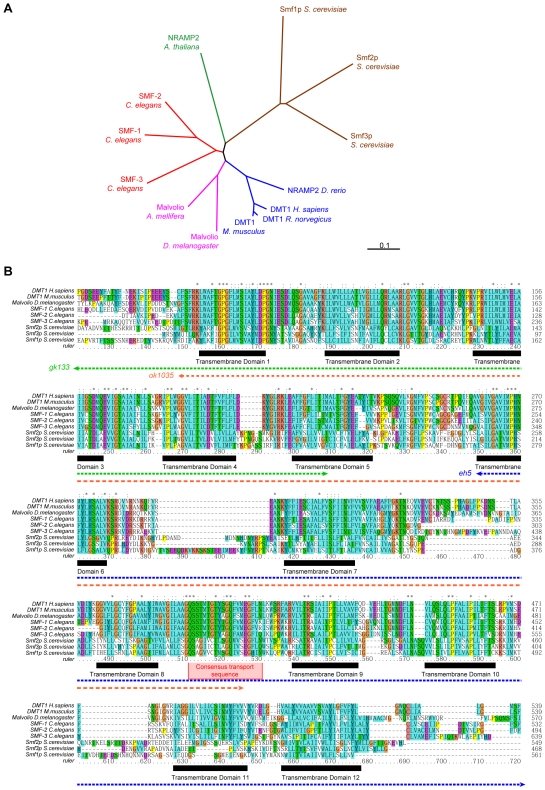
The *C. elegans* genome encodes 3 SMF transporters orthologous to the plant, fungi and animal DMT protein family. (A) Unrooted phylogenetic tree of a subset of eukaryotic members of the DMT family of transporters. *C. elegans* SMF proteins are more closely related to the animal than to the fungus or plant orthologues. (B) Multiple alignment of animal DMT1 orthologues. The 12-transmembrane domain topology of vertebrate DMT1 is conserved in *C. elegans* SMF proteins (black boxes), as well as the consensus transport sequence (red box). Dotted arrows indicate regions of the proteins affected by the deletion alleles *eh5*, *gk113*, and *ok1035* of *smf-1*, *2*, and 3 respectively. Amino acids with similar biochemical properties are highlighted with the same color. * represent residues conserved in all aligned sequences, : corresponds to highly conserved residues and. to less conserved residues.

*The* C. elegans *genome encodes 3 DMT1 orthologues: SMF-1, SMF-2, and SMF-3.* A search on the *C. elegans* genome using the human DMT1 protein sequence revealed three loci encoding DMT1 orthologues: *Y69A2AR.4/smf-3* on chromosome IV, and *K11G12.3/smf-2* and *K11G12.4/smf-1* on X chromosome. All three genes encode proteins with a high degree of conservation (Figure 1). Phylogenetic analysis placed them closer to their insects and vertebrates orthologues than plant and yeast divalent-metal transporters (Fig. 1A). A multiple alignment of the *C. elegans* SMF-1, SMF-2, SMF-3 with their insect, vertebrates and yeast orthologues confirmed this analysis. All three *C. elegans* SMF exhibit a consensus transport sequence (CTS) quasi-identical to their vertebrate orthologues and 12 transmembrane domains (TMD), unlike the yeast SMF, in which the 11^th^ TMD is absent and the CTS is conserved at 70% (Fig. 1B).

**Figure 2:**
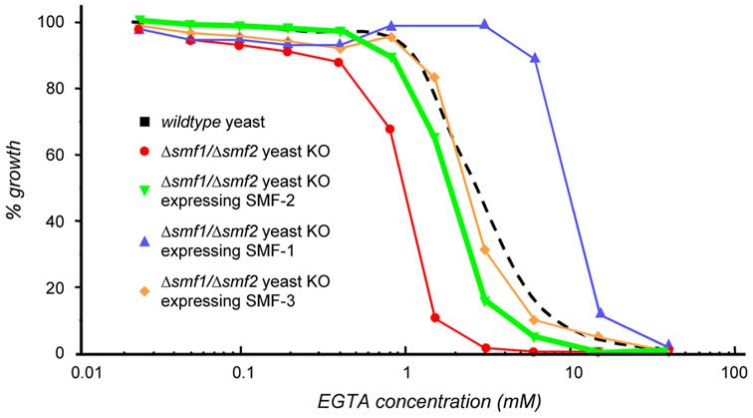
*C. elegans* SMF transporters rescue EGTA sensitivity of yeast Δ*Smf1*+Δ*Smf-2* mutant. *S. cerevisiae* double-mutant *Smf1*Δ+*Smf-2*Δ is hypersensitive to exposure to the divalent cation chelator EGTA (red), when compared to wildtype (dotted black line). Transvections of *C. elegans smf-1* (blue), *smf-2* (green) or *smf-3* (orange) cDNA rescue the double-mutant hypersensitivity to EGTA.

SMF-1, SMF-2 *and* SMF-3 *rescue the sensitivity to EGTA of the yeast ΔSMF1+2 mutant.* In the yeast *S. cerevisiae*, Smf1p and Smf2p are divalent-metal transporters that can function in Mn uptake. Double mutants for *ΔSMF1+2* are hypersensitive to the divalent-metal ion scavenger ethylene glycol tetra-acetic acid (EGTA). To assay the molecular function of *C. elegans* SMF-1, -2 and -3 proteins as divalent-metal transporters, we transvected the cDNA encoding each of the *C. elegans* proteins into the *ΔSMF1+2* yeast mutant, and tested these transgenic strains for hypersensitivity to EGTA. All three *C. elegans* SMF were able to rescue the hypersensitivity to EGTA of the *ΔSMF1+2* mutants (Fig. 2). Moreover, the efficacy of the rescue was at least equivalent to the rescue conferred by transvection of the mouse NRAMP2 cDNA construct [58]. Noticeably, the most potent effect was obtained with *smf-1* cDNA, which led to a hyper-resistant phenotype to EGTA. These results indicate that *C. elegans* SMF-1, SMF-2 and SMF-3 are functionally similar to their yeast and mammal orthologues and are likely involved in divalent-metal ion transport.

**Figure 3:**
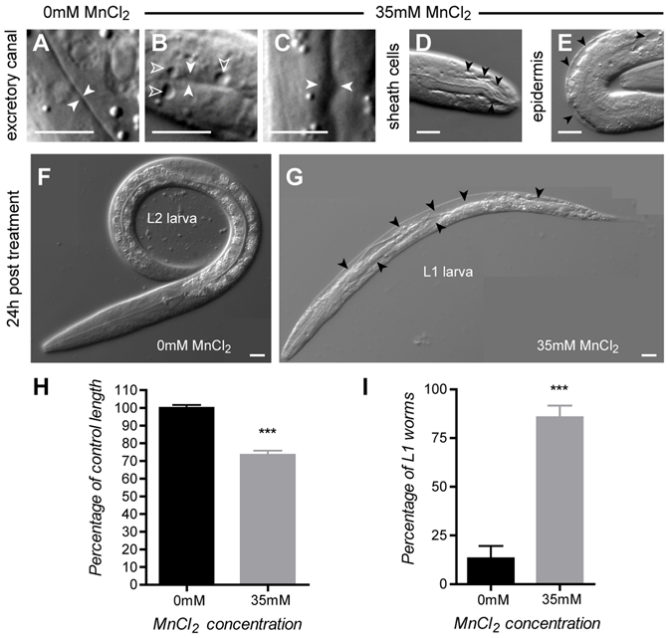
Mn exposure leads to severe osmoregulation defects and developmental delay. (A) Excretory canal in a control wild type L1 larva (solid white arrow heads). (B, C) Enlargement of the excretory canal in L1 larvae acutely exposed to 35 mM MnCl_2_, after 24 h (solid white arrowheads) is associated with vacuolization (hollow arrowheads). Vacuoles are also observed in the sheath cells of the chemosensory organs (D) and in the epidermis (E). (F) Control larva 24 h after 0 mM MnCl_2_ treatment. (G) Dying vacuolated (black arrowheads) larva 24 h after 35 mM MnCl_2_ exposure. (H) worms exposed to 35 mM MnCl_2_ (grey) are about 30% shorter than control animals (black). I, most larvae exposed to 35 mM MnCl_2_ are still in L1 stage at 24 h post-treatment when control animals are L2. Error bars represent SEM, *** p<0.001, scale bars are 5 µm.

**Figure 4:**
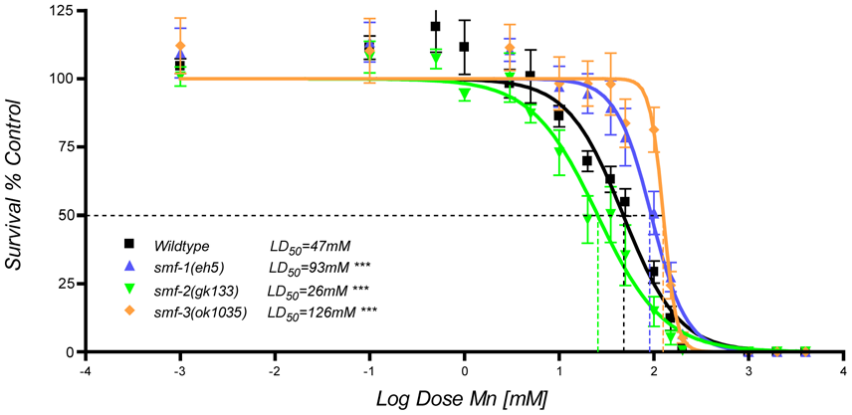
Dose-response lethality curves reveal a differential sensitivity to Mn exposure for *smf* mutants compared to wild type worms. Upon 30 min exposure to MnCl_2_ as L1 larvae the lethal concentration 50 (LD_50_) at which half of the worms were dead at 24 h, was 47 mM for wildtype worms (black, N = 12), 93 mM for *smf-1* mutants (blue, N = 6), 26 mM for *smf-2* mutants (green, N = 7), and 126 mM for *smf-3* mutants (orange, N = 5). Error bars represent SEM, *** p<0.001.

*Mn exposure can induce lethal osmoregulation defects and a developmental delay in wild type worms.* Acute exposure (30 minutes) of wild-type *C. elegans* to Mn was lethal at concentrations greater than 10 mM. Shortly after treatment and dependent upon the exposure level, a fraction of the worms displayed vacuoles in the main epithelia: epidermis, excretory cell and gut, tissues whose integrity is essential for worm survival (Fig. 3). Loss of the excretory cell was the likely cause of death, since dying worms exhibited the characteristic rod-like phenotype (Fig. 3F&G) inherent to excretory-cell defective mutants [64] and excretory-cell ablated worms [65]. Surviving worms at 24 h were about 70% shorter (Fig. 3H) and displayed an obvious developmental delay when compared with control animals. Twenty-four hours post-treatment 83% of survivors exposed to a 35 mM 30 min Mn treatment were still at the L1 stage, compared to 13% in the control group (Fig. 3I). The lethal concentration 50% (LD_50_ at which half the worms are dead 24 hours after treatment) was 47 mM for the wild-type Bristol N2 strain (Fig. 4).

*The deletion-mutants* smf-1(eh5) *and* smf-3(ok1035) *are hyper-resistant to Mn exposure.* In rodents, Mn taken up by ingestion or inhalation is transported across the epithelial membrane via DMT1 [66]. If DMT1 orthologues are responsible for Mn uptake in *C. elegans*, the loss of function or down-regulation of these transporters should reduce Mn sensitivity. To determine if *smf-1*, *smf-2* and *smf-3* are involved in Mn uptake and toxicity in the worm, we made use of available deletion-mutants for each of the three genes. Mutant *smf-1(eh5)*, *smf-2(gk133)* and *smf-3(ok1035)* strains were obtained from the Caenorhabditis Genetic Center (CGC). Under standard culture conditions, none of them displayed any obvious abnormal phenotype with respect to body morphology, development, growth, reproduction or behavior (data not shown). The *smf-1* deletion *eh5* resulted in a truncated SMF-1 protein containing only the 6 first transmembrane domains and predicted to be devoid of Mn transport activity (Fig. 1B). When exposed to Mn, *smf-1(eh5)* mutants were twice as resistant as wild-type, with a LD_50_ = 94 mM (Fig. 4). The *smf-3* deletion *ok1035* removes about 1,8kb in a region encompassing exons 4 to 8. It leads to a loss of at least two of the transmembrane domains 1 to 8, preventing the resulting truncated protein from adopting its functional topology. In addition, the likely loss of TMD6 impairs divalent metal (Me^2+^) transport as this domain was proven essential for H^+^/Me^2+^ symporter activity [47]. In support of this assumption, the *smf-3(ok1035)* mutant displayed the highest resistance to Mn exposure with a LD_50_ = 126 mM (Fig. 4). The hyper-resistance to Mn exposure exhibited by both *smf-1(eh5)* and *smf-3(ok1035)* mutants suggests that the Mn-induced toxicity observed in wild type worms was at least, in part, mediated through Mn uptake by the DMT1-like isoforms. A reasonable explanation for this observation is that Mn uptake in these mutants is impaired and that both SMF-3 and SMF-1 are required for normal and optimal Mn uptake.

*The deletion mutant* smf-2(gk133) *is hypersensitive to Mn treatment.* Concerning *smf-2*, the *gk133* deletion affects the last hundred bases of its promoter up to the third intron, which likely results in the production of a defective protein, lacking the N-terminal sequence up to the 5^th^ TMD (Fig. 1B). Unlike *smf-1(eh5)* and *smf-3(ok1035)*, *smf-2(gk133)* mutant display a significant hypersensitivity to Mn exposure with a LD_50_ = 26 mM (Fig. 4), suggesting a protective role for SMF-2 against Mn toxicity.

**Figure 5:**
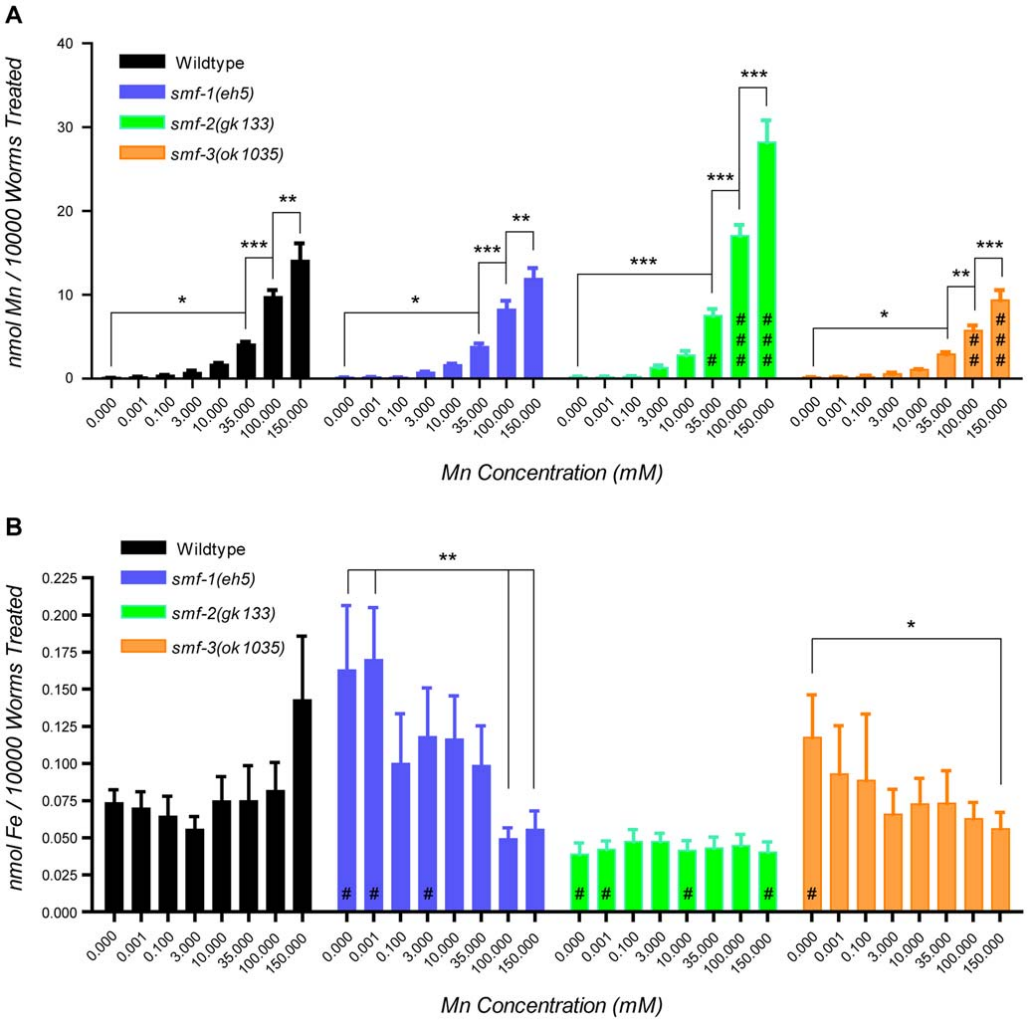
Variations in Mn and Fe content in *smf* mutant worms upon Mn exposure. (A) WT and *smf* mutants take up Mn in a dose-dependent manner. *smf-2(gk133)* (green) takes up significantly more Mn than WT (black) and other mutant worms following exposure to 35 (# p<0.05), 100 (## p<0.001) and 150 mM (### p<0.001) MnCl_2_. *smf-3(ok1035)* (orange) mutants take up significantly less Mn than other worms at 100 (## p<0.01) and 150 mM (### p<0.001). (B) Fe content varies differentially in *smf* mutants and WT upon Mn exposure. *smf-2(gk133)* (green) display significantly lower Fe levels (# p<0.05), while *smf-1(eh5)* and *smf-3(ok1035)* mutants show higher Fe levels (# p<0.05) than WT in absence of Mn treatment (0 mM), and at very low Mn concentration for *smf-1(eh5)* (0.001 mM). Error bars represent SEM. While # designate significant differences between genetic backgrounds exposed to the same manganese dose, * indicate significant differences between exposure doses within the same *C. elegans* strain: #/* p<0.05. ##/** p<0.01, ###/*** p<0.001.

*smf-3(ok1035) mutants take up less Mn while smf-2(gk133) mutants take up more.* To confirm that the SMF proteins are involved in Mn uptake, following acute Mn exposure, we collected wild-type, *smf-1(eh5)*, *smf-2(gk133)*, and *smf-3(ok1035)* worms, and processed them for Atomic Absorption Spectroscopy (AAS) analysis to measure their Mn content. All four strains showed dose-dependent increases in Mn content (Fig. 5A). *smf-1(eh5)* mutants accumulated less Mn than wildtype animals, but the trend was not significant at any of the tested doses. Strikingly, *smf-2(gk133)* accumulated significantly more Mn at 35 mM (p<0.05), 100 mM (p<0.001) and 150 mM (p<0.001) than any other strain, while *smf-3(ok1035)* mutants accumulated significantly less Mn than the other strains, both at 100 mM (p<0.001) and 150 mM (p<0.001).

*smf-1(eh5) mutants exhibit a Mn-dependent decrease in Fe content while smf-2(gk133) mutants are naturally depleted in Fe.* In addition to Mn^2+^, DMT1 can transport a wide range of metallic cations including Zn^2+^, Pb^2+^, Cd^2+^, Ni^2+^, Co^2+^, and Fe^2+^ [67]. It has been shown in rodents and cell cultures that Mn and Fe compete for transport via DMT1 [66], [68]. In particular, high intracellular Fe levels lead to reduced Mn intake in rats [69]. To take into account a potential influence of Fe levels on Mn content, we analyzed Fe levels in parallel to Mn content. This analysis revealed that *smf-2(gk133)* mutants have a significantly lower Fe content than the other strains, which was not affected by Mn dose (Fig. 5B). Wildtype Fe levels were not significantly impacted by 0 to 100 mM Mn exposures, but showed an increase at 150 mM. Upon transient Mn depletion, *smf-1(eh5)* and *smf-3(ok1035)* mutants displayed higher Fe levels, but they underwent a gradual Fe depletion with increasing Mn doses (Fig. 5B). *smf-1(eh5)* Fe content significantly dropped below WT levels when exposed to 150 mM Mn, suggesting a role for SMF-1 in Fe uptake at high Mn doses.

*SMF-1 and SMF-3 expression partially overlaps in the main epithelia while SMF-2 is restricted to minor epithelial tissues.*

To understand better the differential roles of the *smf* genes in *C. elegans*, we studied their expression pattern. We generated transgenic strains expressing the green fluorescent protein (GFP) under the control of *smf-1*, *smf-2* or *smf-3* promoters (*smf-1::GFP*, *smf-2::GFP*, *smf-3::GFP*), as well as strains expressing GFP-tagged SMF-1, SMF-2 and SMF-3 (*SMF-1::GFP*, *SMF-2::GFP*, *SMF-3::GFP)*. In all cases the GFP signal was detected from late embryogenesis to adult stage, with generally higher expression levels in young larvae (L1 stage). None of the transgenic strains obtained showed any reproductive, developmental, behavioral or morphological defects, or intracellular GFP aggregates, thus establishing that the transgenes did not adversely affect the worms' physiology and that their expression levels were likely within the physiological range.

**Figure 6:**
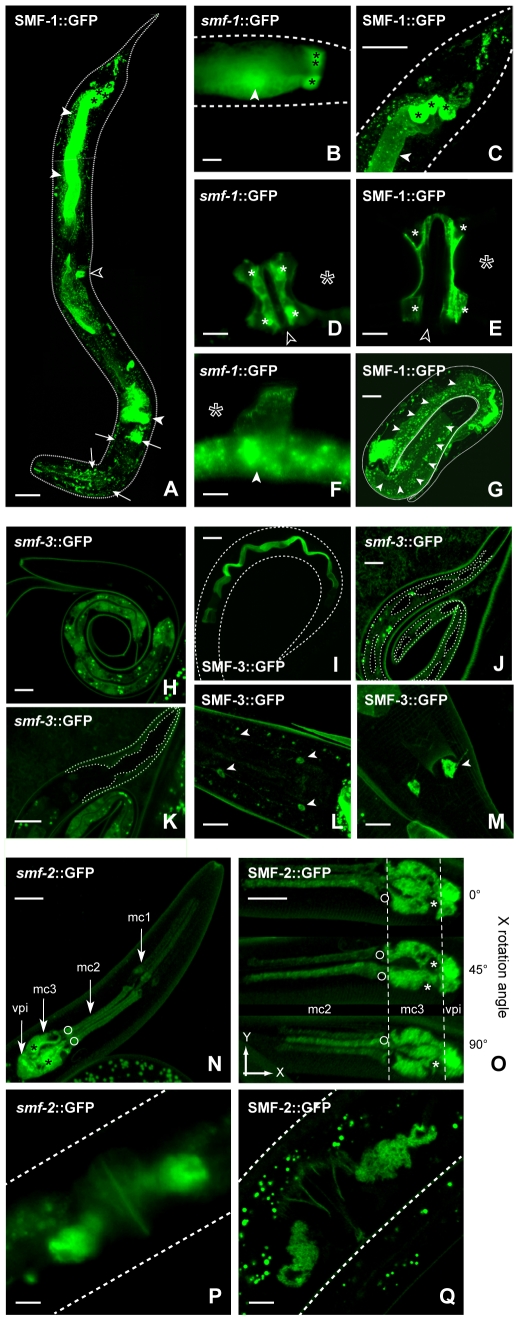
Expression pattern analysis of *C. elegans smf* genes. A, SMF-1::GFP strongly localizes to the anterior and posterior intestine (solid white arrowheads), to the anchor cell (hollow arrowhead) and to head neurons (white arrows). *smf-1::*GFP and SMF-1::GFP reveal expression of *smf-1* gene in rectal gland cells (B,C, black asterisks), in the uterus (uv1, uv2, utse syncytium, D,E, solid white asterisks) as well as in the adult spermatheca (F) and the L1 hyp7 epidermis (G, white arrowheads). Dotted lines outline the cuticle of the worm. Hollow asterisks indicate position of fertilized embryos. Hollow arrowheads indicate position of the vulva. *smf-3* is mainly expressed in the intestine as revealed by *smf-3*::GFP (H) and SMF-3::GFP (I), in the major epidermis hyp7 (J, dotted line) and head epidermis hyp1-6 (K, dotted line), and in head (L) and tail neurons (M). An antero-posterior gradient of *smf-2* expression is noticeable in the 9 marginal epithelial cells of the pharynx (mc1, mc2, mc3) and the 6 vpi cells of the pharyngeo-intestinal valve (N and O). Fainter expression is consistently observed in the proximal gonad (P, Q). Scale bars are 5 µm.

Both *smf-1::GFP* and *SMF-1::GFP* were prominently expressed in the anterior and posterior intestine and associated gland cells (Fig. 6A, 6B, 6C). A strong expression was observed in the anchor cell (AC) during larval life and in the adult proximal uterus resulting from the fusion of AC with uv1, uv2 and utse cells (Fig. 6A, 6D, 6E). GFP signal was also consistently seen in the adult spermatheca (Fig. 6F). Fainter expression was observed in the major epidermis hyp7, in the pharyngeal muscles, and in a subset of anterior sensory neurons, ring neurons, and posterior-head neurons (Fig. 6A, 6G). SMF-3::GFP was mostly observed all along the intestine, with a weaker expression in the most proximal and distal regions (Fig. 6H, 6I). A weak epidermal expression in hyp1-6, hyp7 and hyp8-12, and in head and tail neurons was also seen (Fig. 6J, 6K, 6L, 6M). In contrast to the broad SMF-1::GFP and SMF-3::GFP expression patterns, SMF-2::GFP was mostly restricted to the mc1, mc2, mc3 epithelial cells of the pharynx and the pharyngeal-intestinal valve cells vpi1-6, displaying an anterior-posterior expression gradient (Fig. 6N, 6O). SMF-2 was also observed in the gonad sheath cells at the adult stage (Fig. 6P, 6Q).

**Figure 7:**
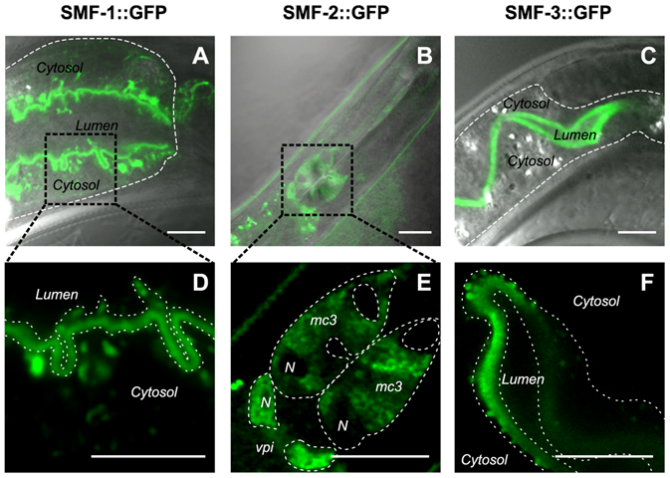
Subcellular localization of SMF::GFP reporters. SMF-1::GFP mostly localizes to the apical plasma membrane of the intestine and sub-apical compartments (A, D, dotted line in A underlines the basolateral membrane of the intestine, dotted lines in D delimit its apical plasma membrane). SMF-2::GFP is seen in cytoplasmic organelles in mc and vpi cells (E, cell plasma membranes are marked by a dotted line, *N* indicate the position of the nuclei). SMF-3::GFP is mainly restricted to the apical plasma membrane of the intestine and apical vesicular organelles (C, F, dotted line in C underlines the basolateral membrane of the intestine, dotted lines in D delimit its apical plasma membrane). Scale bars are 5 µm.

*SMF-1 and SMF-3 are apically localized while SMF-2 is mainly cytoplasmic.* The full-length SMF-1::GFP and SMF-3::GFP allowed us to observe the intracellular localization of SMF-1 and SMF-3. In vertebrates, the DMT1 isoforms are mostly localized at the apical side in intestinal, rectal and kidney epithelial cells [66]. In agreement, SMF-1::GFP and SMF-3::GFP were localized at the apical plasma membrane in all epithelia in which they were expressed (Fig. 7A, 7B). Conversely, SMF-2::GFP was associated with intracellular cytoplasmic compartments and to a lesser extent to the apical membrane of the mc epithelial cells (Fig. 7C).

**Figure 8:**
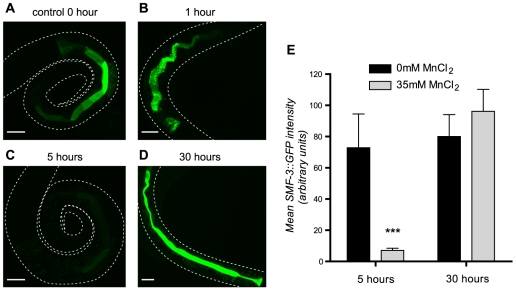
SMF-3::GFP is down-regulated upon Mn exposure. SMF-3::GFP signal is strongly detected at the apical plasma membrane prior Mn treatment (A). After 1 hour of exposure to 35 mM MnCl2, SMF-3-GFP localizes to sub-apical vesicular compartments (B). C, SMF-3::GFP signal is strongly decreased at 5 hours post-treatment. After a day of recovery, SMF-3::GFP expression returns to control levels and SMF-3::GFP relocates to the apical plasma membrane (D). Scale bars are 5 µm. (E) quantification of apical plasma-membrane SMF-3::GFP in the whole intestine after 5 and 30 h of exposure in control and treated animals. While # designate significant differences between genetic backgrounds exposed to the same manganese dose, * indicate significant differences between exposure doses within the same *C. elegans* strain: #/* p<0.05. ##/** p<0.01, ###/p<0.001.

*Variation in SMF protein levels/localization upon Mn exposure.* Studies in vertebrates and yeast showed that DMT1 and DMT1-like isoforms are differentially regulated by metals, such as Fe and Mn [66]. In particular, modulation of expression and protein levels by Fe has been extensively studied [66]. In vertebrates, the *DMT1* gene encodes two to four isoforms, defining two functional types which depend upon the presence of an Iron-Responsive Element (IRE) in their 3′UTR (+IRE isoform) or its absence (-IRE isoform) [70]. A consensus sequence for this IRE has been identified in several proteins involved in Fe metabolism and regulation [71]. Yeast studies also showed differential regulation of *Smf* genes upon Mn and Fe exposure or depletion [55]. More specifically, in Mn-replete conditions, Smf1p and Smf2p are targeted to the vacuole for degradation, but they accumulate at the plasma membrane and in intracellular vesicles upon Mn depletion [56], [72], [73]. Unlike Smf1p and Smf2p, which are regulated by Mn at a post-translational level, Sm3p is regulated by Fe at a transcriptional level [56]. Given the impact of metal cation levels (Fe and Mn) on DMT1 isoform expression and localization in both vertebrate and fungus models, we addressed the possibility that *C. elegans* SMF activity may also change with environmental Mn concentration. We used *C. elegans* strains transgenic for the SMF::GFP reporters to follow DMT1-like protein expression and localization changes upon Mn exposure. We performed the same L1 acute treatment protocol as for our Mn toxicity and Mn content measurements, and collected worms at 1, 5 or 30 hours post-treatments for image analysis. SMF-1::GFP and SMF-2::GFP expressing worms did not show any obvious changes in either expression levels or intracellular localization. However, 1 hour post-treatment, SMF-3::GFP was translocated to apical vesicular compartments in the L1 intestine (Fig. 8A, 8B); at 5 hours the intestinal GFP signal was considerably reduced; it was not solely restricted to the apical plasma membrane, showing also cytoplasmic expression (Fig. 8C). This phenomenon was reversible, since at 30 hours post-exposure, both the expression levels and the subcellular localization of SMF-3::GFP returned to normal (Fig. 8D). These observations are supported by quantifications of SMF-3::GFP fluorescence at 5 and 30 hours, showing a significant decrease of SMF-3::GFP levels at 5 hours, but not at 30 hours post-treatment (Fig. 8E). This result suggests that SMF-3 function is regulated by Mn levels and further supports a major role for SMF-3 in Mn uptake.

**Figure 9:**
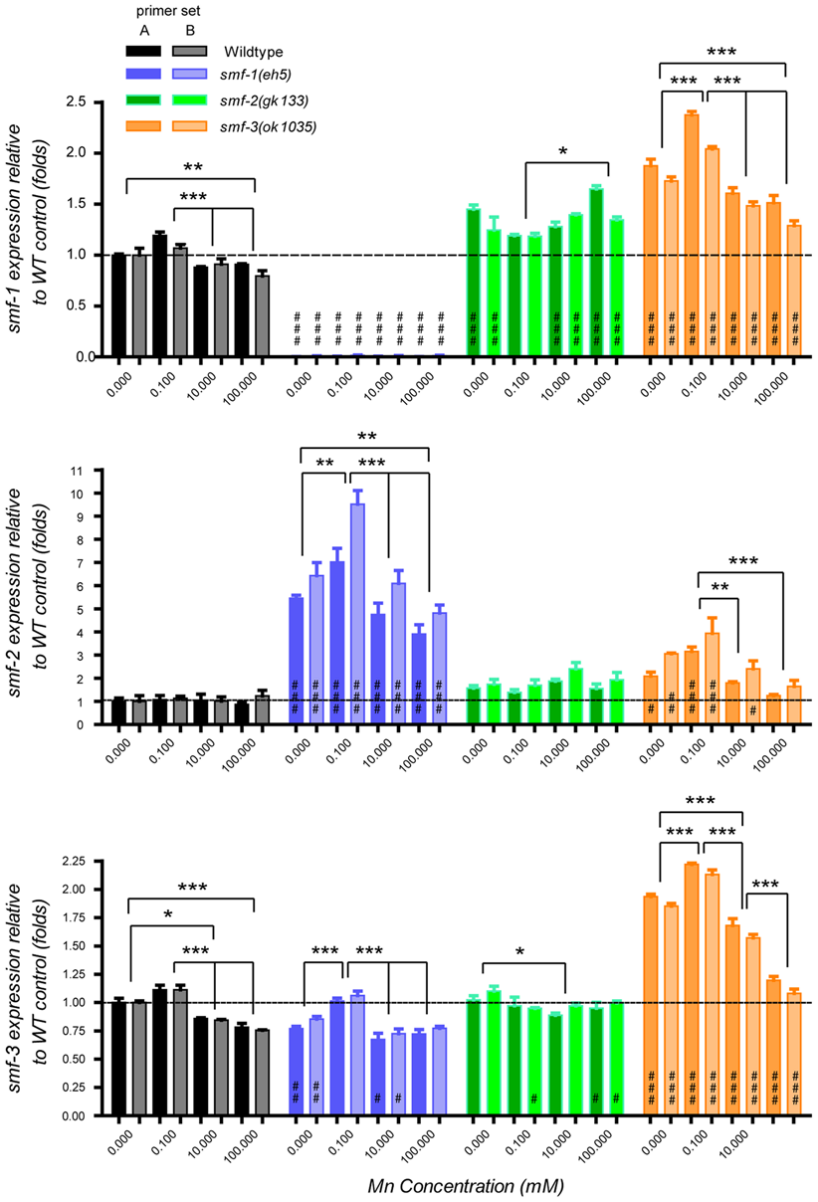
Genotype and Mn exposure influence on *smf* gene expression. Both independent primer sets corresponding to the 3′ end of the cDNA and used to assess *smf* gene expression (set A bright colors, and set B faded colors) give consistent results (A, B, C). *smf-1* and *smf-3* mRNA levels display similar variations, increasing at low Mn exposure (0.1 mM) and decreasing upon high Mn concentrations (10 mM and 100 mM) in WT, *smf-1* and *smf-3* mutants (A, C, black, blue, orange). *smf-2* mRNA levels follow the same tendency in *smf-1* and *smf-3* mutants but are not affected by Mn exposure in WT (B). Independent of Mn exposure, *smf-1(eh5)* mutant is characterized by a strong up-regulation of *smf-2* expression (B, blue). Compared to other genotypes, *smf* expression in *smf-2(gk133)* does not appear to correlate with Mn exposure (A, B, C, green). The *smf-3(ok1035)* mutant exhibits higher *smf* gene expression levels regardless of Mn exposure dose (A, B, C, orange). While # designate significant differences between genetic backgrounds exposed to the same manganese dose, * indicate significant differences between exposure doses within the same *C. elegans* strain: #/* p<0.05. ##/** p<0.01, ###/p<0.001. Displayed significance levels between treatments reflect the weakest significance score obtained between both primer sets.

*Variation in smf gene expression by RT-PCR.* Given the fact that *C. elegans* expresses DMT1-like isoforms with partially overlapping expression patterns, we examined if they displayed some functional redundancy and if their relative expression levels were dependent on each other and on metal levels. We also wondered whether SMF-3::GFP down-regulation upon Mn exposure could be explained by transcriptional inhibition. To address these issues we conducted quantitative real-time reverse-PCR assays (QRT-PCR) on wild-type, *smf-1(eh5)*, *smf-2(gk133)*, and *smf-3(ok1035)* worms acutely exposed to 0, 1, 10, 100 mM of MnCl_2_. Both primers sets used to monitor *smf* gene transcriptional levels showed that *smf-1* and sm*f-3* were up-regulated at 0.1 mM of Mn and down-regulated at 10 and 100 mM Mn in WT and *smf* mutants, but not in *smf-2(gk133)* (Fig. 9A, 9B, 9C). As expected, *smf-1* expression was undetectable in *smf-1(eh5)*, since the *smf-1* primers were chosen within the deleted region in *smf-1(eh5)* (Fig. 9A). In *smf-1(eh5)* and *smf-3(ok1035)* mutants, *smf-2* was also up-regulated at 0.1 mM of Mn and down-regulated at 10 and 100 mM Mn, while in WT, *smf-2* expression was not affected by any of the Mn levels (Fig. 9B).

Noticeably, *smf-2* was strongly overexpressed in *smf-1(eh5)* (Fig. 9B). In *smf-3(ok1035)*, all *smf* genes were found overexpressed compared to WT, especially *smf-3* (Fig. 9C), implying that a functional SMF-3 is required to maintain normally low *smf* gene expression. Lastly, *smf* gene expression was not influenced by Mn exposure in the *smf-2(gk133)* genetic background (Fig. 9A, 9B, 9C). This observation supports a role for SMF-2 in *smf* gene regulation upon changes in environmental Mn status.

## Discussion

We identified three new functional members of the DMT1 family in *C. elegans*. All three rescued the yeast *ΔSMF1+2* EGTA sensitivity, and revealed important for metal homeostasis in the worm, with very little functional overlap. SMF-1 and SMF-3 were broadly expressed in C. *elegans* epithelia; they were required for Mn uptake and toxicity, and exhibited the classical apical localization of DMT1. Conversely, SMF-2 expression was essentially restricted to specialized pharyngeal cells, where it was predominantly cytoplasmic, and to endow partial protection against Mn-induced toxicity. SMF-1 and SMF-2 were both required for normal Fe intake, SMF-2 being necessary for Mn-dependent Fe content regulation. Mn exposure revealed influential on transcriptional regulation of the three *smf* genes, but only affected the post-translational regulation of SMF-3. Our results unravel a complex metal-regulation system involving 3 distinct DMTs coordinately regulated by metal levels, and bear interesting implications on the modulation of the vertebrate DMT activities carried out by NRAMP1 and DMT1 isoforms.

*SMF-3 regulates and is specifically regulated by Mn.* In addition to expression and localization data, Mn content measurement and dose-response lethality curves in *smf* mutants clearly indicate that SMF-3 is a major Mn transporter in the worm, which is required for Mn-induced toxicity. *smf-3(ok1035)* is the mutant most resistant to acute Mn exposure (Fig. 4). SMF-3 is expressed in the epidermis and located all along the intestine, where most Mn absorption likely occurs (Fig. 6). SMF-3 sub-cellular localization is apical, consistent with a role in Mn uptake (Fig. 7). *smf-3(ok1035)* is the only mutant that displays a significant decrease in Mn content upon MnCl_2_ treatment (Fig. 5), implying that SMF-1 or SMF-2 cannot compensate for SMF-3 depletion. Moreover, SMF-3 levels and apical localization are strongly decreased 5 hours after toxic Mn exposure and are restored after a 24 hour recovery period (Fig. 8). Finally, QRT-PCR revealed a decrease in *smf-3* mRNA levels upon Mn exposure at 10 and 100 mM (Fig. 9). These observations support the notion that *smf-3* is down-regulated both transcriptionally and post-translationally upon Mn exposure, which limits toxic accumulation of Mn in the worm. After a day of recovery allowing for the excretion of Mn accumulated upon previous exposure, SMF-3 levels return back to normal levels, permitting nutritional divalent cation uptake. Our experiments support a regulation of SMF-3 levels at the post-translational level, by relocating and degrading SMF-3. The translocation of SMF-3 likely to endosomal compartments in response to excessive Mn exposure can be related to the vacuolar targeting of Smf1p and Smf2p in Mn-replete conditions in yeast [56], [73], [74]. In porcine kidney cells, (-IRE)-DMT1 isoforms have been shown to be more rapidly and efficiently internalized upon metal exposure when compared to (+IRE)-DMT1 isoforms [39]. Although we did not find any typical IRE sequence in any of the three *smf* 3′UTR sequences (data not shown), we observed a drastic internalization of SMF-3 upon Mn treatment, but not of SMF-1 or SMF-2. Additionally, DMT1 has the highest affinity for Mn^2+^ [67] and SMF-3 is the *C. elegans* orthologue that is responsible for most Mn uptake. Hence, in *C. elegans*, SMF-3 may be the functional counterpart of the vertebrate -IRE forms of DMT1.

*An atypical role for SMF-2 in Mn homeostasis. smf-2* is located downstream of *smf-1* on the X chromosome. Due to the absence of any splice acceptor site in the *smf-2* sequence and based on our GFP-reporter analysis, it is unlikely that *smf-1* and *smf-2* belong to the same operon. Indeed, both exhibited very distinct expression patterns (Fig. 6) and mutations in *smf-1* or *smf-2* have opposite effects on Mn sensitivity (Fig. 2). The high degree of conservation between both genes suggests that they result from a duplication that happened early in the *Caenorhabditis* lineage, since it is also found on the *C. briggsae* and *C. remanei* X chromosomes (http://dev.wormbase.org/db/seq/gbrowse_syn/pecan/?search_src=elegansname=chrX%3A6716267..6721098). *smf-2(gk133)* mutants exhibited hypersensitivity to Mn exposure and a higher Mn content than wild type worms (Fig. 2, 5), indicating that SMF-2 partially protects against Mn exposure, which may not be solely explained by a role in Mn uptake. The intestinal expression of SMF-1 and SMF-3 from late embryogenesis to adulthood strongly supports their involvement in metal uptake, while SMF-2 expression and subcellular localization in the pharyngeal epithelial cells is intriguing, since these cells are not known to be involved in nutrient or toxicant uptake (http://www.wormatlas.org/handbook/alimentary/alimentary1.htm).

**Figure 10:**
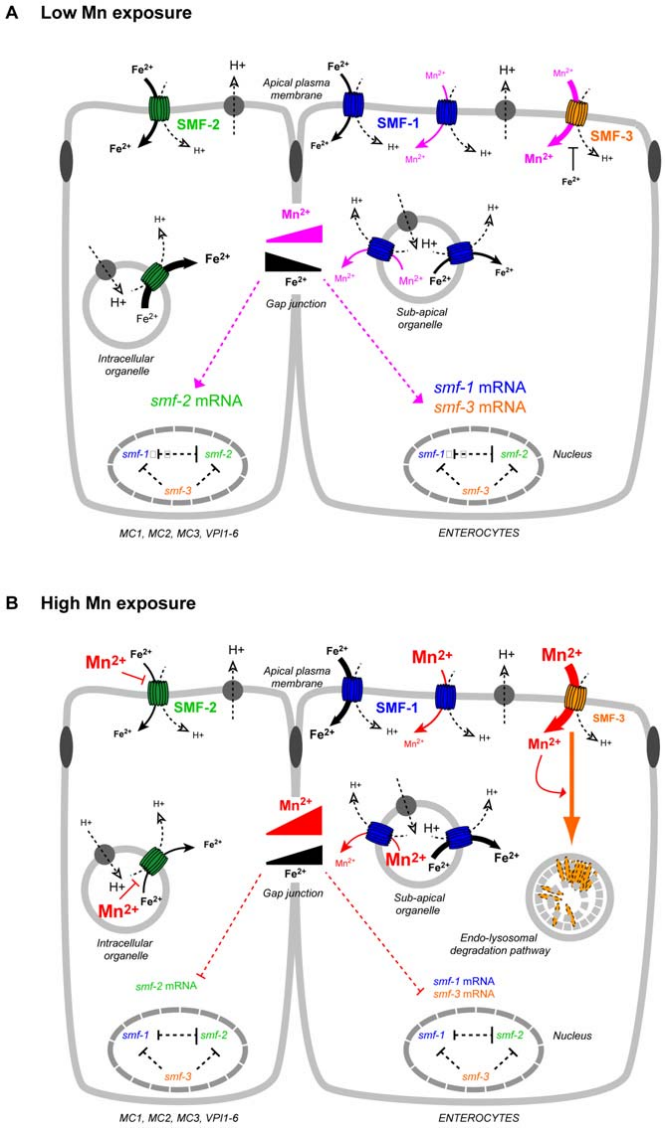
Working model for Mn and Fe uptake by SMF transporters in *C. elegans*. A: Regulation of Mn, Fe contents and SMF transporters upon low Mn exposure (0.001 mM to 3 mM), which is believed to be beneficial for the worm physiology [92]. B: Regulation of Mn, Fe contents and SMF transporters upon high Mn exposure (50 mM to 150 mM), which was shown to be toxic (Fig. 3, 4). We propose that SMF-3* is the main transporter responsible for Mn uptake (A), and that it is degraded upon exposure to high Mn concentrations (B). Since high Fe content limit Mn uptake, SMF-3 may be inhibited by intracellular Fe (A). SMF-1 would be involved in Mn uptake to a lesser extent, and together with SMF-2*, would be responsible for Fe uptake. Upon Low Mn exposure SMF-2 would be mostly required for Fe uptake (A), whereas upon high Mn exposure, SMF-2 would be inhibited and SMF-1 would partially compensate for Fe uptake (B). In the case of SMF-2 and SMF-1, metal uptake could essentially take place in acidified endosomal compartments, as SMF-2 is mainly cytoplasmic and SMF-1 is detected in sub-apical compartments. Gap-junction communications between pharyngeal epithelia, vpi cells and intestinal cells permit Mn^2+^ and Fe^2+^ to diffuse distant from their site of uptake, allowing metal-dependent regulation of *smf* mRNA stability or transcription. A prediction of our model is that the Fe gradient established by SMF-2 activity would be reversed upon high Mn exposure (B), and could constitute the signal for *smf* expression regulation. Since basal *smf* mRNA levels depend on the integrity of each *smf* genomic sequence (Fig. 9), transcriptions of *smf* genes are assumed to be interdependent, maybe because they require a common transcription factor. * SMF-2 might transport Mn and SMF-3 might transport Fe, but these possibilities are not explored in this model. The size of the text reflects the concentration of the species.

SMF-2 may rather be involved in the regulation of Mn toxicity, either by allowing its excretion, its sequestration, or the modulation of Mn uptake via the other DMT1-like isoforms, or a combination of the above. Because of the structural and functional similarities between SMF-3, SMF-1 and SMF-2, we favor the idea that pharyngeal SMF-2 takes up metallic cations, allowing for the overall regulation of Mn content. Several hypotheses can be formulated. First, SMF-2 could work as a sensor of environmental Mn levels, and a downstream signaling pathway would either impact Mn uptake via SMF-3 and SMF-1 or Mn excretion via as of yet uncharacterized transporters. The anterior-posterior gradient of SMF-2 expression in the pharynx would provide a differential mechanism to accurately estimate metal concentrations. Second, SMF-2-driven inhibition of Mn uptake could involve modulation of the pharyngeal pumping. The marginal cells of the pharynx, which express SMF-2, are electrically coupled to the pharyngeal muscles by gap-junctions [75]. In mammalian cells, high Mn concentrations have been shown to impact cardiomyocyte contractility by inhibiting the Ca^2+^-ATPase [76], [77]. Considering the structural and functional similarities between the *C. elegans* pharynx and the vertebrate heart [78], Mn uptake via SMF-2 in marginal cells might impact pharyngeal muscle activity. Decrease in pharyngeal pumping would result in reduced nutrient and Mn intake. This could also account for the slow growth of Mn-treated animals. Third, our Fe content analysis suggests that SMF-2 is an important Fe transporter. The lack of SMF-2 leads to lower Fe levels in the *smf-2(gk133)* worms (Fig. 5B), which would allow for increased Mn uptake by SMF-3 or SMF-1 (Fig. 5A). Moreover, Fe content is high at low Mn exposure, and drops at high Mn exposure in *smf-1(eh5)* and *smf-3(ok1035)* mutants, while it is constant in *smf-2(gk133)* mutants (Fig. 5B). The *smf* gene expression also follows this trend. It suggests that SMF-2 is required for Mn-dependent variations in Fe content and *smf* gene expression. SMF-2 is active in a different cell type as SMF-1 and SMF-3 (Fig. 4), and those cells are connected through gap-junctions. To reconcile these data, we propose that SMF-2-dependent Fe-uptake is inhibited at high Mn doses, leading to a Fe-depletion in the pharynx and a reversed Fe gradient across the animal. In turn this Fe imbalance, together with increased Mn content would lead to the down-regulation of *smf* genes in a SMF-2-dependent manner (Fig. 10B).

Based on SMF-2::GFP subcellular localization (Fig. 7), which appears to be in puncta in the cytoplasm, but not in the nucleus, SMF-2 could be present in most membranous intracellular organelles of the endo-lysosomal pathway. Its activity could be increased by the V-ATPase-driven acidification of those compartments, similarly to DMT1 in endosomes and NRAMP1 in phagosomes [33], leading to metal transport to the cytosol. On the one hand, generation of double-transgenic *C. elegans* strains to conduct colocalization studies with lyzosomal (LAMP-1 and LAMP-2) and endosomal (RAB-5, RAB-7) markers would be beneficial in determining if the puncta seen in Fig. 7 correspond to endo-lyzosomal compartments. On the other hand, colocalization with an essential V-ATPase subunit, such as VHA-8 [89], and observation of orange acridine (pH-sensitive vital dye) staining in SMF-2-positive puncta and measurement of *C. elegans* Fe content upon variation in environmental pH, could be used to study the pH-dependency of SMF-2 function.

*SMF-1: minor in Mn physiology, major in Fe homeostasis?* Though expression pattern, sub-cellular localization and mutant analysis might suggest an important role for SMF-1 in Mn^2+^ uptake, Mn content measurements failed to show a significant difference between wild type and *smf-1(eh5)* mutant worms. Thus, a difference in Mn uptake may not account for their resistance to Mn exposure. Several hypotheses may be advanced to explain this apparent discrepancy. First, SMF-1 may mediate Mn toxicity subsequent to Mn uptake at the apical membrane of the intestine, transporting Mn from endocytic compartments to the cytosol. In absence of SMF-1, endocytic Mn content would remain the same, but Mn would not be transported across the membrane to reach the cytosol and trigger toxicity (Fig. 10). The presence of SMF-1 in intracellular vesicular organelles supports this idea (Fig. 7). When located at the apical plasma membrane, SMF-1 would then be largely inactive; being activated upon acidification of the endocytic vesicles by the vacuolar-ATPase [33], [48], [79]. Second, SMF-1′s contribution to Mn uptake may be minor compared to SMF-3 and Mn content measurements would not be able to reflect the relatively limited, yet physiologically significant variations in Mn content. This alternative explanation is logical within the context of the DMT family, in which different isoforms have been shown to transport distinct cations with variable efficiencies. Hence, the vertebrate NRAMP1 is a critical Fe transporter in macrophages [80], [81], [82], and has a poor affinity for Mn compared to DMT1 [67]. SMF-1 may thus be the counterpart of NRAMP1 in the worm, being predominantly required for Fe uptake. Additionally, *smf-1(eh5*) is the only mutant which showed a significant Mn-dependent depletion of Fe content, despite the overexpression of *smf-2* in this context. It indicates that SMF-1 is required to take up or conserve Fe at high Mn concentrations, and may be responsible for Fe homeostasis (Fig. 10). Third, the QRT-PCR experiments revealed that the *smf-1(eh5*) mutant exhibits a higher *smf-2* expression level than wildtype, *smf-2(gk133)* and *smf-3(ok1035)* worms. If as mentioned previously SMF-2 is protective against Mn toxicity, the resistant phenotype of the *smf-1(eh5*) mutant may reflect the overexpression of SMF-2. In this case, SMF-1 would not necessarily be involved in Mn transport. Fourth, the previous hypotheses are not mutually exclusive. Lastly, SMF-1 is able to rescue EGTA sensitivity in yeast beyond WT level (Fig. 2). This better rescue could be due to a higher transgene copy number or a better stability of the SMF-1 product in the yeast, resulting in higher SMF-1 levels and a hypermorphic phenotype. However, it could also indicate that this transporter has a broader range of substrates than SMF-2 and SMF-3 and may be functionally closer to a canonical DMT.

*New insights for the study of the eukaryotic family of DMT1-related transporters.* Our analysis of the *C. elegans* DMTs reveals that they are closely related structurally and functionally to their vertebrate orthologues. SMF-1 and SMF-3 are apically localized in epidermal and intestinal cells, consistent with observations about DMT1 distribution and sub-cellular localization in vertebrates [47], [85], [86]. Moreover, DMT1 proteins transport divalent metallic cations via coupling with the proton gradient [47] in part generated by the vacuolar-ATPase apically localized both in vertebrates and in *C. elegans* epithelia [66], [89], [90]. Interestingly, the three *C. elegans* isoforms display distinct expression levels and contribute differentially to Mn and Fe physiology, similarly to the +IRE, -IRE forms of DMT1 and NRAMP1 in vertebrates. The fact that SMF-2 antagonizes SMF-3 could be connected to the suspected antiporter activity of NRAMP1 relatively to the symporter activity of DMT1. NRAMP1 was proposed to be extruding metals against the proton gradient [91]. This activity would rely on specific residues located before the transmembrane domains (TMD) 9 and 12, which differ between NRAMP1 and DMT1. In particular, in mammals, NRAMP1 exhibits a basic residue K389 instead of a polar residue N403 for DMT1, at the end of the CTS. Interestingly, our multiple-alignment reveals that all other DMT1 orthologues including Malvolio, SMF-3 as well as yeast Smf1p, Smf2p and Smf3p display a polar residue (N or Q) at this position, while SMF-1 and SMF-2 show a basic residue (R or K) similarly to NRAMP1 (Fig. 1). SMF-2 also seemed to limit Mn accumulation. However, unlike NRAMP1, SMF-1 and SMF-2 efficiently rescue the yeast *ΔSMF1+2* EGTA sensitivity. Moreover, their expression in the proximal digestive system argues against an antiporter activity that would result in metal extrusion. Therefore, it would be very interesting to test the ability of SMF-1 and SMF-2 to rescue the heavy metal ion stress in *ΔBSD2/ΔRER1* yeast mutants. Ultimately the symporter or antiporter activity of DMTs may depend on the metal ions and their concentrations on both sides of the membrane. Environmental, cytosolic and organelle metal concentrations in yeast, oocytes, macrophages or distinct *C. elegans* cell types may vary enough for DMTs to function differently depending on the context. Another point is that the specific expression patterns of SMF-1, SMF-2 and SMF-3 remained the same through the worm's post-embryonic life, implying that the cell-specific expression levels of *smf* genes are embryonically determined, further variations resulting from changes in metal exposure and physiology.

Analysis of our results allows us to propose a working model for Mn and Fe uptake regulation in *C. elegans* (Fig. 10). We favor the notion that metal ion gradients play the key role in the regulation of expression, sub-cellular localization and activity of DMTs in *C. elegans*. To further test this idea, fluorescent ferrochromes could be used to visualize *in vivo* the Fe gradient upon various Mn exposures. To yield further information on the metal-transport abilities of the various DMTs from worm to human, one could perform a systematical comparative analysis of DMT family member biochemical properties in yeast using complementation rescue of *ΔBSD2/ΔRER1* and *ΔSMF1+2* mutants, coupled to an *in vivo* study using metal-exposed transgenic *C. elegans* expressing tissue-specific chimerical DMT transgenes. Such a dual approach should help solve the paradox concerning the antiporter activity of NRAMP1 orthologues, and the effect of metal gradients on DMT activity.

To dissect out the relative contributions of each *smf* gene in metal physiology in *C. elegans*, the generation of double or triple *smf* mutants would also be of great interest. However generation of the *smf-1;smf-2* double mutant and the triple mutant *smf-1;smf-2;smf-3* would require a new deletion mutant in which both *smf-1* and *smf-2* expressions are lost, since the short genetic distance between both genes would make recombination events between *smf-1(eh5)* and *smf-2(gk133)* very unlikely. In addition, considering that *smf-2* is strongly up-regulated in *smf-1(eh5)* (Fig. 9), it is possible that the *eh5* deletion, by bringing *smf-1* promoting or enhancing sequences closer to the *smf-2* gene, affects its natural expression. This potential issue should be carefully considered before further usage of *smf-1(eh5)* to generate double-mutants. Combining RNAi and single mutants could be another way to address the redundancy issue, with the limitation that RNAi may not be fully penetrant or specific enough to affect single isoforms.

*Relevance of our* C. elegans *model to study Mn toxicity and related diseases.* Mn exposure has long been suspected to be responsible for IPD cases. The commonalities between manganism and PD suggest that both pathologies, at least in part, rely on shared genetic networks and molecular mechanisms. Due to the increasing incidence of PD, notably in most populous countries [23], there is growing interest in identifying contributing genetic and environmental factors. In this context, development of amenable genetic models to study gene-environment interactions is essential. Our work corroborates the utility of *C. elegans* as an appropriate complementary whole-animal model to decipher molecular mechanisms and genetic predisposition to metal induced toxicity. First, we showed that *C. elegans* takes up Mn readily and is sensitive to Mn exposure, exhibiting developmental delay, excretory cell and osmoregulatory defects which can be related to kidney and bladder defects observed in rodents [83], [84] and man [44]. The high Mn concentrations used in our study (when compared to cell-culture studies in which the LD_50_ approximates 1 mM for 24 h exposures) likely reflect three characteristics of the model. *C. elegans* is sheathed with a cuticle impermeable to ions and only few orifices would physically allow metal ion uptake, namely the mouth, the rectum, the pore cell of the excretory system and the chemosensory organs. In support of this, the Mn content measured by atomic absorption spetrophotometry (AAS) in wild-type worms treated at the LD_50_ (47 mM) corresponded to an effective dose of 12.5 mM. Another point to consider is that the worms in the present study were acutely treated (30 min) in contrast to most cell culture studies that involve more protracted exposures (>24 h). It is also likely that the worm, naturally living in soil, where ion concentrations fluctuate greatly and rapidly, may be physiologically more robust than mammalian cells. Second, our functional analysis of the SMF transporters in the yeast (Fig. 2) together with the metal content measurements in wild type and *smf* mutant worms (Fig. 5A), show that the three *C. elegans* DMTs are involved in metal uptake regulation. Third, two of these transporters, SMF-1 and SMF-3 are mostly expressed in the intestine, a portal for Mn and Fe uptake both in worms and vertebrates. SMF-1 and SMF-3 are also apically localized in epidermal and intestinal cells, consistent with observations about DMT1 distribution and sub-cellular localization in vertebrates [47], [85], [86]. Fourth, several facts inherent to the regulation of DMTs by Mn or Fe and vice-versa previously described in mammalian models also hold true in *C. elegans*. For example, we showed that low Mn exposures (0.1 mM) tend to increase and high concentrations (100 mM) to decrease *smf* gene expression (Fig. 9). SMF-2 was required in this regulation process as Mn concentration-dependent effects are not observed in the *smf-2(gk133)* mutant (Fig. 5, 9). Fifth, Fe and Mn tissue levels are interdependent and depend on environmental concentrations. In the *smf-2(gk133)* mutant, low Fe content is associated with an increased ability to take up Mn. In *smf-1(eh5)*, Fe content decreases with increasing Mn exposure (Fig. 5). Last, we found that SMF-3 N-terminal sequence contains a putative Mitochondrial Targeting Sequence (MTS, probability 93.66% given by MitoProt, http://ihg2.helmholtz-muenchen.de/ihg/mitoprot.html): MPRVHRQSRWNSVSFSGFFLQISGIKPRF. Our analysis of SMF-3::GFP sub-cellular localization did not reveal any epithelial mitochondrial targeting *in vivo*. However, it could happen in tissues that were not investigated in this study such as neurons, or in situations that were not covered by our experimental conditions. If it was confirmed, a mitochondrial targeting of DMT1 orthologues would mean that their activity could directly impact mitochondrial function and potentially apoptosis, in particular in neurodegenerative processes [87], [88].

Taken together, our data suggest that Mn uptake and toxicity mechanisms involving DMTs are conserved from nematodes to man. Because other genetically amenable invertebrates such as *D. melanogatser* only express one DMT (*Malvolio*, *Mvl*), and given the large panel of currently available techniques on the nematode (toxicology, molecular biology, forward and reverse genetics, confocal and electron-microscopy, biochemistry), *C. elegans* provides a convenient *in vivo* platform to further investigate metal toxicity and related neurodegenerative disorders in which DMT1-related transporters are suspected to play an important role [87], [88].
